# The Furosemide Stress Test: A Dynamic Tool for Predicting Acute Kidney Injury Progression in Critical Care Medicine

**DOI:** 10.3390/jcm14082595

**Published:** 2025-04-10

**Authors:** Luigi La Via, Giuseppe Cuttone, Nicola Sinatra, Maurizio Giuseppe Abrignani, Giulio Geraci, Giovanni Ippati, Francesca Maria Rubulotta

**Affiliations:** 1 Department of Anesthesia and Intensive Care 1, University Hospital Policlinico “G. Rodolico–San Marco”, 95123 Catania, Italy; francesca.rubulotta@unict.it; 2 Trauma Center Unit, “Villa Sofia-Cervello” Hospital, 90146 Palermo, Italy; giuseppe.cuttone@hotmail.it; 3 Nephrology and Dialysis Unit, “Paolo Borsellino” Hospital, 91025 Marsala, Italy; sinatra.nicola@libero.it; 4 Cardiology Unit, “Paolo Borsellino” Hospital, 91025 Marsala, Italy; maur.abri60@gmail.com; 5 Faculty of Medicine and Surgery, Kore University, 94100 Enna, Italy; giulio.geraci@unikore.it; 6 Department of Anesthesia and Intensive Care, “S.A. Abate” Hospital, 91016 Erice, Italy; ippatigiovanni@gmail.com; 7 Department of General Surgery and Medical Surgical Specialties, University of Catania, 95124 Catania, Italy; 8 The International Women in Intensive and Critical Care Network IWIN Foundation, 94011 Sicily, Italy

**Keywords:** acute kidney injury, furosemide stress test, critical care, renal function, prognostic tool, nephrology

## Abstract

Acute kidney injury (AKI) remains a significant challenge in critical care medicine, affecting up to 50% of intensive care unit patients with substantial mortality rates. While traditional approaches to AKI assessment rely on static measurements like serum creatinine and urine output, the furosemide stress test (FST) has emerged as a dynamic functional tool for evaluating renal tubular function and predicting AKI progression. This comprehensive review examines the historical development, physiological basis, technical aspects, and clinical applications of FST in various patient populations. Originally developed and validated in 2013, FST has demonstrated superior predictive capabilities for AKI progression and the need for renal replacement therapy compared to conventional biomarkers. The test’s mechanism relies on assessing the kidney’s response to a standardized furosemide challenge, providing insights into both the structural integrity and functional reserve of the renal tubular system. Standardized protocols have been established for different clinical scenarios, though implementation challenges remain, including timing considerations, patient selection, and resource requirements. FST has shown utility in critical care, post-cardiac surgery, sepsis-associated AKI, and heart failure settings. Recent developments include integration with artificial intelligence, personalized medicine approaches, and combination with novel biomarkers. While limitations exist, including contraindications and technical challenges, ongoing research continues to refine protocols and expand applications. This review highlights FST’s role as a valuable prognostic tool in modern AKI management and discusses future directions, including automated monitoring systems, protocol standardization efforts, and potential applications in different patient populations.

## 1. Introduction

Acute kidney injury (AKI) remains a significant challenge in critical care medicine, affecting up to 50% of intensive care unit patients and carrying mortality rates that can exceed 50% in severe cases [1,2]. Over the past decades, our understanding of AKI has evolved from a simple binary condition to a complex syndrome with various phenotypes, trajectories, and outcomes [3]. Traditional approaches to AKI prognostication have relied heavily on static measurements such as serum creatinine and urine output, as defined by the KDIGO criteria/guidelines [4]. However, these conventional markers have significant limitations, including a delayed recognition of kidney injury and a limited ability to predict progression to severe AKI or the need for renal replacement therapy (RRT) [5,6]. The search for more accurate prognostic tools has led to the development of various biomarkers, including Neutrophil Gelatinase-Associated Lipocalin (NGAL), Interleukin-18 (IL-18), and Kidney Injury Molecule-1 (KIM-1) [7]. While these novel biomarkers have shown promise, their clinical utility has been limited by cost, availability, and the time required for results [8]. Furthermore, they provide only a snapshot of kidney injury without reflecting the kidney’s functional reserve or recovery potential [9]. This gap in dynamic assessment capabilities has driven the need for functional tests that can evaluate renal stress responses in real-time. The furosemide stress test (FST) emerged as a practical tool that leverages the physiological response to loop diuretics to assess tubular function and predict AKI progression [10,11]. By measuring the kidney’s response to a standardized furosemide challenge, clinicians can gain insights into both the structural integrity and functional reserve of the renal tubular system [12]. The FST represents a paradigm shift in AKI assessment, moving from static measurements to dynamic functional testing [13]. This approach aligns with the growing recognition that AKI is not merely a change in creatinine or urine output but rather a complex syndrome requiring sophisticated prognostic tools for optimal patient care [14,15].

## 2. Historical Background

**Figure 1:**
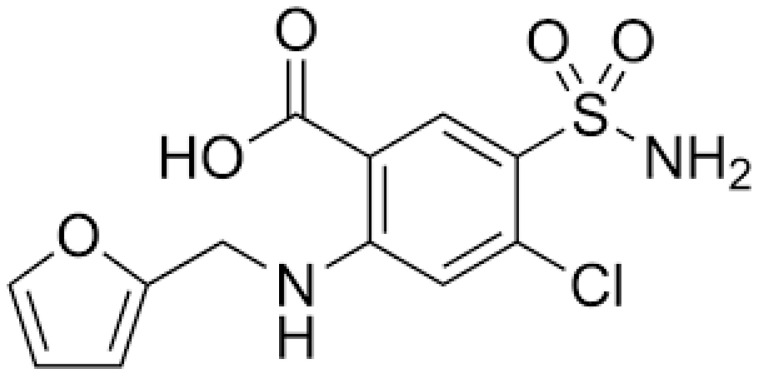
Chemical structure of furosemide.

The concept of using furosemide as a diagnostic tool emerged from clinical observations in the 1970s, when researchers first noted that the response to loop diuretics could provide insights into renal function [16]. However, it was not until the early 2000s that systematic approaches to using furosemide as a predictive tool began to emerge, driven by the need for better prognostic indicators in AKI [17]. The modern FST was first formally developed and described by Chawla and colleagues in 2013 [10]. Their landmark study established the foundational protocol, which involved administering a standardized dose of furosemide (Figure 1) (1.0 or 1.5 mg/kg) to patients with early AKI and measuring their urinary output response over the subsequent hours.

This initial work demonstrated remarkable predictive capability for progression to advanced-stage AKI, with the sensitivity and specificity exceeding those of contemporary biomarkers [18]. The validation phase of the FST followed multiple paths. Single-center studies first confirmed the reproducibility of the original findings [19], while subsequent multicenter investigations established the test’s broader applicability [12]. A pivotal multicenter study in 2015 validated the FST’s ability to predict progression to stage 3 AKI and the need for renal replacement therapy, showing an area under the receiver operating characteristic curve of 0.87 [11]. International validation studies emerged from 2016 onwards, demonstrating the test’s utility across different patient populations and healthcare settings [20]. These studies were particularly significant in establishing FST’s performance in diverse clinical contexts, from cardiac surgery to sepsis-induced AKI [13]. The test’s predictive value was found to be consistent across different ethnic groups and healthcare systems, supporting its robust nature [21]. By 2018, meta-analyses began to aggregate the growing body of evidence, confirming the FST’s role as a reliable prognostic tool [22]. These analyses highlighted not only the test’s predictive accuracy but also its cost-effectiveness and practical advantages over traditional biomarker-based approaches [23]. The standardization of the protocol and establishment of clear cut-off values for interpretation were crucial developments during this period [24]. The historical development of the FST represents a shift from empirical observations to evidence-based practice, culminating in its current status as a validated tool for AKI prognostication [25]. This evolution parallels the broader transformation in critical care nephrology, where dynamic functional assessments are increasingly valued over static measurements [26].

## 3. Physiological Mechanisms

The physiological principles underlying the FST are rooted in the complex interactions between loop diuretics and renal tubular function. Furosemide, a loop diuretic, exerts its effect by inhibiting the Na^+^/K^+^/2Cl^−^ cotransporter (NKCC2) in the thick ascending limb of the loop of Henle [27]. Understanding this mechanism is crucial for appreciating the test’s diagnostic and prognostic value in AKI. In normal renal physiology, furosemide reaches the tubular lumen through active secretion via the organic anion transport system in the proximal tubule [28]. This process requires adequate renal blood flow and functioning proximal tubular cells. Once secreted, furosemide binds to the luminal surface of the NKCC2 transporter, inhibiting sodium reabsorption and increasing urinary sodium and water excretion [29]. During AKI, several pathophysiological changes can affect the response to furosemide. Reduced renal blood flow impairs drug delivery to the proximal tubule, while tubular injury disrupts both the secretion of furosemide and its interaction with the NKCC2 transporter [30]. The severity of these impairments correlates with the extent of kidney injury, providing the theoretical basis for the FST’s predictive capabilities [31]. The test’s ability to predict AKI progression relies on assessing the kidney’s functional reserve. A robust urine output response indicates preserved tubular function and adequate renal perfusion, suggesting a higher likelihood of recovery [11]. Conversely, a poor response often reflects more severe structural damage or significant hemodynamic compromise, predicting a higher risk of AKI progression [32]. Recent research has elucidated additional mechanisms that influence FST results. These include the role of renal autoregulation, intrarenal hemodynamics, and the impact of inflammatory mediators on tubular function [33]. Studies have also revealed that the test response correlates with markers of tubular injury and repair, providing insights into the biological basis of its prognostic accuracy [34]. Understanding the timing of the FST is crucial, as the test’s performance varies depending on the phase of AKI. Early administration allows the assessment of renal reserve before extensive structural damage occurs, while maintaining adequate intravascular volume is essential to ensure valid results [35]. The physiological response to the test also varies with different AKI etiologies, reflecting the diverse pathogenic mechanisms involved [36]. Recent molecular studies have demonstrated that the FST response correlates with the expression of injury biomarkers and cellular stress responses [37]. This relationship provides a molecular basis for the test’s ability to predict outcomes and suggests potential applications in personalizing AKI management [38,39,40].

## 4. Clinical Considerations: Protocol, Timing, and Dosing

The effective implementation of the FST requires careful attention to protocol standardization, appropriate timing, and precise dosing considerations. These technical aspects are crucial for achieving reliable and reproducible results across different clinical settings [10].

### 4.1. Protocol

The standardized FST protocol begins with patient assessment to ensure appropriate candidate selection. Key prerequisites include euvolemic status, hemodynamic stability, and the absence of contraindications to loop diuretics [41]. The protocol mandates careful documentation of baseline parameters, including vital signs, fluid status, and recent urine output [42]. Standard dosing follows a weight-based approach, with furosemide-naïve patients receiving 1.0–1.5 mg/kg, while patients on chronic loop diuretics require 1.5–2.0 mg/kg [43]. The medication should be administered intravenously over 20–30 min to minimize ototoxicity risk. The monitoring protocol requires hourly urine output measurement for 6 h, along with regular vital sign assessment, fluid balance tracking, and serum electrolyte monitoring [44].

### 4.2. Timing

The optimal timing for FST administration has been extensively studied. The test is most informative when performed early in the course of AKI, typically within 24–48 h of diagnosis [12]. Evidence suggests that earlier testing provides better predictive value for AKI progression [19]. Critical timing factors encompass the stage of AKI (ideally stage 1 or early stage 2), time from ICU admission, relationship to nephrotoxic exposures, and the achievement of hemodynamic stability [20].

### 4.3. Dosing

Several factors influence dosing decisions, including prior loop diuretic exposure, baseline renal function, body composition, concurrent medications, and underlying comorbidities [45]. Special considerations apply to specific patient populations, as elderly patients may require dose adjustment, while obesity requires the careful consideration of dosing weight. Heart failure patients often need higher doses, and patients with hypoalbuminemia may have altered drug distribution [38,46].

To ensure reliable results, standardized protocols should incorporate clear documentation requirements, response criteria definitions, safety parameters, stop criteria, and volume management guidelines [1,47]. The test has several absolute contraindications, including severe hypovolemia, allergy to sulfa drugs, and severe electrolyte disorders. Relative contraindications comprise unstable hemodynamics, active bleeding, and severe acid-base disturbances [48,49]. Successful implementation requires comprehensive staff training and competency assessment, clear documentation tools, response algorithms, and quality monitoring processes [50]. The standardization of these technical aspects has contributed significantly to the FST’s reliability as a prognostic tool in AKI management [51]. Ongoing refinements in protocol design continue to enhance the test’s utility across different clinical scenarios [52].

## 5. Possible Applications

The FST has demonstrated significant utility across various clinical settings and patient populations, with substantial evidence supporting its prognostic value in different contexts [53]. Understanding these diverse applications helps clinicians optimize the test’s use in specific clinical scenarios (Table 1).

Studies examining long-term outcomes have revealed that FST results correlate not only with short-term renal outcomes but also with longer-term survival and renal recovery [48]. This association persists even after adjusting for conventional risk factors and severity of illness [66]. Economic analyses have supported the cost-effectiveness of FST implementation in appropriate clinical settings, particularly when used to guide early intervention and resource allocation [67]. The test’s ability to identify patients requiring more intensive monitoring or earlier RRT initiation has demonstrated favorable cost–benefit ratios [68].

## 6. Predictive Performance

The FST has demonstrated significant predictive capabilities when compared to traditional biomarkers and clinical scoring systems in AKI assessment. Understanding these comparative performances is crucial for optimal clinical decision-making [69]. Serum creatinine, the conventional marker for kidney dysfunction, has inherent limitations due to its delayed rise after injury and various confounding factors. Studies have shown that FST outperforms serum creatinine in predicting AKI progression, particularly in the early stages of kidney injury [70]. The test demonstrates superior sensitivity and specificity in identifying patients at risk for requiring renal replacement therapy (RRT) [7]. When compared to newer biomarkers such as NGAL, KIM-1, and IL-18, FST has shown comparable or superior predictive value [71]. Research indicates that FST provides more rapid results and maintains accuracy across various clinical scenarios [72]. The test’s performance remains robust even in situations where biomarker levels might be influenced by concurrent conditions such as sepsis or inflammation [73]. Clinical scoring systems like APACHE II, SOFA, and RIFLE criteria have been traditionally used for risk stratification in AKI. Studies demonstrate that FST provides additional predictive value when combined with these scoring systems [74]. The integration of FST results with established scoring methods has shown improved accuracy in predicting adverse outcomes [75]. A significant advantage of FST over both conventional and novel biomarkers is its ability to provide dynamic functional assessment. While most biomarkers offer static measurements, FST provides real-time information about renal functional reserve and recovery potential [76]. This dynamic assessment capability has proven particularly valuable in time-sensitive clinical decision-making [38]. Economic evaluations have demonstrated favorable cost-effectiveness ratios for FST compared to novel biomarker panels [77]. The test’s relatively low cost and widespread availability make it an attractive option, particularly in resource-limited settings [15]. In post-surgical settings, FST has shown superior predictive accuracy compared to traditional markers for post-operative AKI progression [37]. Similarly, in sepsis-associated AKI, the test maintains its predictive value while some biomarkers show variable performance [78]. Recent research has explored the synergistic potential of combining FST with various biomarkers. These studies indicate that integrated approaches may offer enhanced predictive accuracy [5]. The combination of FST with selected biomarkers has demonstrated improved risk stratification capabilities [79]. Despite its strong predictive performance, FST has certain limitations compared to other assessment methods. These include contraindications in specific patient populations and the need for the careful timing of administration [80]. Understanding these limitations is crucial for appropriate test utilization [81]. Emerging research focuses on developing standardized protocols that combine FST with other predictive tools. These integrated approaches aim to optimize risk stratification and guide therapeutic interventions more effectively [82]. Studies have also explored machine learning algorithms that incorporate FST results with other clinical parameters for enhanced predictive accuracy [83]. The comprehensive evaluation of FST’s predictive performance continues to demonstrate its value as a practical and reliable tool in AKI assessment. Its ability to complement and sometimes surpass traditional biomarkers and scoring systems makes it an essential component of modern AKI management strategies [24].

## 7. Limitations and Practical Challenges

While the FST has demonstrated significant utility in AKI assessment, several important limitations and practical challenges must be considered for its optimal implementation in clinical practice [84]. The test’s reliability can be compromised in certain physiological states. Patients with severe volume depletion, hemodynamic instability, or those requiring high-dose vasopressor support may demonstrate altered responses to FST, potentially leading to false results [45]. Additionally, underlying chronic kidney disease can affect the interpretation of FST results, necessitating the careful consideration of baseline renal function [85], particularly given that risk factors and progression patterns in chronic kidney disease follow different trajectories [86]. The optimal timing for FST administration remains a subject of debate. Early administration may miss evolving kidney injury, while delayed testing might result in missed opportunities for early intervention [49]. The test’s performance can also be affected by the timing of previous diuretic administration, requiring the careful coordination of medication schedules [2]. Accurate urine output measurement is crucial for FST interpretation, but this can be challenging in certain clinical settings. Issues with catheter positioning, proper collection techniques, and measurement accuracy can affect result reliability. Furthermore, the standardization of testing conditions across different clinical environments presents ongoing challenges [87]. Several absolute and relative contraindications limit FST applicability. Patients with allergies to loop diuretics, severe hypotension, or specific cardiac conditions may not be suitable candidates [88]. The risk of further volume depletion in vulnerable patients necessitates careful patient selection and monitoring [89]. The implementation of FST requires appropriate infrastructure and staff training. Continuous monitoring capabilities, precise urine output measurement systems, and proper documentation protocols are essential but may not be universally available [1]. The need for dedicated nursing attention during the test period can strain resources in busy clinical units [90]. Result interpretation can be complicated by various factors including concurrent medications, underlying disease states, and variations in patient characteristics [91]. The lack of universally standardized cut-off values for different patient populations adds complexity to result interpretation [92]. Despite growing evidence supporting FST utility, variations in testing protocols exist across institutions. Differences in furosemide dosing, the timing of administration, and response measurement criteria can affect result comparability and generalizability [47]. The need for standardized protocols remains a significant challenge [93]. Maintaining consistent documentation and quality control measures presents practical challenges. Accurate recording of timing, doses, and responses requires careful attention to detail and standardized documentation processes [94]. Integration with electronic health records systems may present additional technical challenges [37]. While FST is relatively inexpensive compared to novel biomarkers, implementation costs can be significant when considering the entire testing infrastructure and personnel requirements [95]. Cost-effectiveness analyses must account for both direct and indirect expenses associated with test implementation [38]. Ensuring proper staff training and maintaining competency standards presents ongoing challenges. Healthcare providers need specific training in test administration, monitoring, and result interpretation [96]. Regular updates and competency assessments are necessary but resource-intensive [97]. Effective communication among different healthcare team members is crucial for successful FST implementation. Clear protocols for result reporting and action plans based on test outcomes need to be established and consistently followed [97]. Interdepartmental coordination can be particularly challenging in large healthcare systems [98]. Addressing these limitations requires ongoing research and protocol refinement. The development of automated monitoring systems, standardized protocols, and clearer guidelines for specific patient populations may help overcome current challenges [66]. Integration with emerging technologies and artificial intelligence systems may provide solutions to some existing limitations [99]. Understanding these limitations and challenges is essential for healthcare providers implementing FST in clinical practice. The careful consideration of these factors enables more effective test utilization and result interpretation, ultimately leading to improved patient care outcomes [53].

## 8. Future Research

The evolving landscape of AKI management presents numerous opportunities for expanding and refining the applications of the FST. Current research directions and potential developments warrant careful consideration for advancing this diagnostic tool [66].

- Integration with Artificial Intelligence

Machine learning algorithms show promising potential for enhancing FST interpretation and prediction accuracy. By incorporating FST results with other clinical parameters, artificial intelligence systems could provide more precise risk stratification and outcome prediction [100]. The development of predictive models that combine FST data with electronic health record information may improve decision-making processes and patient care optimization [99].

- Personalized Medicine Applications

Research is advancing toward customized FST protocols based on individual patient characteristics. Studies have explored how genetic variants, comorbidity profiles, and demographic factors influence FST responses [101]. This personalized approach could lead to more accurate test interpretation and improved therapeutic decision-making [102].

- Novel Monitoring Technologies

The development of automated and continuous monitoring systems for FST implementation is an active area of research. Advanced biosensors and real-time urine output measurement devices could enhance the accuracy and reliability of test results [103]. Integration with wearable technology might enable the more precise timing and monitoring of patient responses [104].

- Protocol Standardization Efforts

Ongoing research focuses on establishing standardized protocols for different clinical scenarios and patient populations. Multi-center studies have evaluated optimal dosing regimens, timing protocols, and response criteria across various clinical settings [24]. These efforts aim to develop more robust and universally applicable testing guidelines [105].

- Biomarker Integration

The investigation of combined approaches incorporating FST with novel biomarkers shows promise. Research suggests that such integrated protocols might provide a more comprehensive assessment of kidney injury and recovery potential [106]. Studies have explored optimal combinations and timing strategies for multiple diagnostic modalities [15].

- Pediatric Applications

The adaptation of FST protocols for pediatric populations represents an important research direction. Studies have investigated age-specific dosing regimens and response criteria [107]. The development of pediatric-specific guidelines could expand the test’s utility in this vulnerable population [108].

- Point-of-Care Applications

Research into the point-of-care applications of FST could expand its accessibility and utility. The development of simplified protocols suitable for emergency departments and outpatient settings is underway [109]. These adaptations could enable earlier intervention in evolving AKI cases [38].

- Recovery Prediction

The enhanced understanding of FST’s role in predicting renal recovery is emerging. Studies have investigated how test responses correlate with long-term outcomes and recovery patterns [48]. This research could improve prognostication and guide rehabilitation strategies [110].

- Protocol Refinements

The investigation of modified FST protocols continues, including the evaluation of different diuretic agents and administration methods. A study has explored the potential benefits of continuous infusion versus bolus administration [111]. Studies have also assessed the impact of timing variations on test performance [49].

- Cost-Effectiveness Analysis

Comprehensive economic evaluations of FST implementation are ongoing. Studies have examined the cost–benefit ratio of various protocol modifications and implementation strategies [95]. These analyses will help optimize resource allocation and healthcare planning [112].

- Quality Improvement Integration

Studies have investigated how FST can be better integrated into existing quality improvement programs. Research has also focused on developing standardized care bundles that incorporate FST results [97]. These efforts aim to enhance overall AKI management strategies [98].

- Multi-organ Assessment

New research about the FST’s potential role in assessing other organ systems is emerging. Studies suggest possible applications in evaluating cardio-renal interactions and systemic fluid status [113]. These investigations could expand the test’s utility beyond traditional AKI assessment [114].

Future research priorities should also address significant knowledge gaps in FST applications. Topics that are particularly necessary are as follows:

- Studies Examining Special Populations

The primary area requiring investigation is the systematic evaluation of FST performance in special patient populations. Specifically, research is needed to understand test reliability and interpretation in patients with pre-existing chronic kidney disease, fluid overload, and known diuretic resistance. These studies should aim to develop population-specific protocols and adjusted interpretation criteria that account for these underlying conditions.

- Technical Validation Studies

Technical validation studies represent another crucial research direction. These should focus on standardizing FST protocols for patients with altered drug metabolism and developing modified cut-off values for different comorbidity profiles. Additionally, research should examine optimal timing strategies across various clinical scenarios, as the temporal relationship between FST administration and AKI progression remains incompletely understood.

- Integration with Modern Technologies

The integration of FST with modern technologies presents a particularly promising avenue for investigation. Research is needed to develop and validate machine learning algorithms that specifically incorporate FST parameters alongside other clinical data [115]. Studies should explore the potential of artificial intelligence in FST interpretation and investigate automated monitoring systems for real-time FST analysis. Furthermore, research should examine how FST data can be meaningfully integrated with electronic health record predictive models to enhance clinical decision-making.

These knowledge gaps represent critical areas where additional research could significantly enhance our understanding of FST applications and limitations, potentially leading to the more personalized and precise use of this diagnostic tool.

## 9. Conclusions

The future of FST research holds significant promise for improving AKI management. Continued investigation and protocol refinement will likely enhance our understanding and application of this valuable diagnostic tool. Integration with emerging technologies and personalized medicine approaches may further expand its clinical utility and impact on patient outcomes.

## Figures and Tables

**Table 1:** Possible clinical applications of furosemide stress test (FTS). AKI: acute kidney injury; NGAL: Neutrophil Gelatinase-Associated Lipocalin; IL-18: Interleukin-18; KIM-1: Kidney Injury Molecule-1; RRT: renal replacement therapy.

CRITICAL CARE SETTING	Remarkable predictive accuracy for AKI progression and the need for RRT. A poor response to FST correlates strongly with worse outcomes in this population [23]. Superior predictive values compared to conventional clinical parameters and standard AKI biomarkers [19].
CARDIAC SURGERY	Post-cardiac surgery patients represent a distinct population where FST has proven valuable. Early administration of FST in the post-operative period can effectively identify patients at risk for severe AKI [54]; The test’s performance in this setting is particularly notable given the complex pathophysiology of post-cardiac surgery AKI, involving both inflammatory and ischemia–reperfusion mechanisms [55].
SEPSIS-ASSOCIATED AKI	In patients with sepsis-associated AKI, the FST has demonstrated specific utility in predicting renal outcomes. Research indicates that the test maintains its predictive value even in the context of vasopressor support and complex fluid management requirements [56]. The response to FST in septic patients also provides insights into underlying hemodynamic patterns and potential therapeutic responses [57].
HEART FAILURE	Patients with acute decompensated heart failure present unique challenges in AKI assessment. Studies have shown that FST results in this population can help differentiate between primary cardiac and renal pathology [58]. The test’s ability to predict diuretic responsiveness has proven particularly valuable in managing complex cardiorenal interactions [59].
TRANSPLANT RECIPIENS	Useful tool for evaluating graft dysfunction. The test can help differentiate between various causes of early post-transplant dysfunction and guide therapeutic interventions [60]. The test’s performance in this setting has been validated in both living and deceased donor transplant recipients [61].
EMERGENCY DEPARTMENT APPLICATIONS	Recent studies have explored FST utility in emergency department settings, particularly for the risk stratification of patients presenting with early AKI. The test has shown promise in identifying patients who require admission and those who can be safely managed as outpatients [62].
SPECIAL POPULATIONS	The elderly population presents unique considerations in FST interpretation, with evidence suggesting modified response thresholds may be necessary [63] Similarly, in patients with chronic kidney disease, the test’s predictive value remains intact, though interpretation must account for baseline renal function [24].
INTEGRATION WITH BIOMARKERS	Contemporary research has focused on combining FST results with novel biomarkers, demonstrating enhanced predictive accuracy when used in conjunction with molecules such as NGAL, KIM-1, and IL-18 [64]. This integrated approach has shown superior performance compared to either modality alone [65].

## Data Availability

No new data were created for this article.

## Abbreviations

The following abbreviations are used in this manuscript:

FST	Furosemide Stress Test
AKI	Acute Kidney injury
NGAL	Neutrophil Gelatinase-Associated Lipocalin
KIM-1	Kidney Injury Molecule-1
IL-18	Interleukin-18
NKCC2	Na^+^/K^+^/2Cl^−^ cotransporter
RRT	Renal replacement therapy
